# Immune-modulating copper-doped bioactive glass-based injectable hydrogel for periodontitis therapy

**DOI:** 10.3389/fmolb.2026.1843490

**Published:** 2026-09-21

**Authors:** Huiqin lv, Onaza Ali, Ting Tu, Zhe Liu, Yunyan Zhan

**Affiliations:** 1 School of Stomatology, Jiangxi Medical College, Nanchang University, Nanchang, China; 2 Jiangxi Provincial Key Laboratory of Oral Diseases, Nanchang, China; 3 Jiangxi Provincial Clinical Research Center for Oral Diseases, Nanchang, China; 4 School of Biomedical Engineering and Med-X Research Institute, Shanghai Jiao Tong University, Shanghai, China

**Keywords:** bacterial infection, borate ester bonds, copper-doped bioactive glass, immunomodulatory, periodontitis therapy

## Abstract

**Background:**

Periodontitis, a chronic inflammatory disorder driven by microbial dysbiosis and aberrant host immune responses, poses a significant challenge to periodontal tissue regeneration owing to persistent infection and impaired osteogenesis.

**Objective:**

Herein, we engineered an injectable hydrogel (denoted Cu@BG-HA) via borate ester crosslinking of copper-doped mesoporous bioactive glass nanoparticles (Cu@BG, 480 nm) with hyaluronic acid, aiming to concurrently address antibacterial, immunomodulatory, and osteogenic deficits in the periodontal microenvironment.

**Methods:**

The Cu@BG-HA hydrogel was synthesized through dynamic borate ester bond formation between Cu@BG nanoparticles and HA polymer networks. The physicochemical properties, ion release kinetics, and therapeutic efficacy were systematically characterized using *in vitro* models of bacterial infection, macrophage polarization, and osteoblast differentiation.

**Results:**

The Cu@BG-HA hydrogel demonstrated sustained Cu^2+^ release over 24 days, achieving eradication rates of 99.8% ± 3.2% against *Staphylococcus aureus* and 98.6% ± 1.7% against *Porphyromonas gingivalis*. Under lipopolysaccharide/interferon-γ (LPS/IFN-γ) stimulation, the hydrogel effectively orchestrated macrophage phenotype switching from pro-inflammatory M1 toward anti-inflammatory M2 states in RAW264.7 cells. Furthermore, Cu@BG-HA markedly potentiated osteogenic differentiation of MC3T3-E1 pre-osteoblasts, evidenced by 1.8- and 1.7-fold enhancements in alkaline phosphatase activity and mineralized nodule formation, respectively, relative to control conditions.

**Conclusion:**

Collectively, these findings establish Cu@BG-HA as a multifunctional, minimally invasive therapeutic platform that integrates potent antibacterial, immunomodulatory, and pro-osteogenic functionalities. This strategy offers substantial translational potential for periodontitis management and may be extendable to other inflammatory osteolytic pathologies.

## 1 Introduction

Periodontitis is a chronic inflammatory disease characterized by the progressive destruction of the periodontal supporting tissues (Zheng et al., 2023; Li Q. et al., 2024; Hou et al., 2025), including the gingiva, periodontal ligament, cementum, and alveolar bone (Pan Q. et al., 2025; Wang et al., 2023). The pathogenesis of periodontitis is multifactorial and involves a complex interplay between microbial dysbiosis and host immune responses (Hu C. et al., 2024). Dysbiosis of the oral microbiome, particularly the overgrowth of pathogenic bacteria such as *Porphyromonas gingivalis* (Ma et al., 2022), *Staphylococcus aureus* (Wang J. et al., 2025) and *Escherichia coli* (Wu et al., 2021), triggers a cascade of inflammatory reactions that ultimately lead to tissue destruction and bone resorption (Wu et al., 2025). Additionally, the host immune response, often characterized by an imbalance in macrophage polarization between the pro-inflammatory (M1) and anti-inflammatory (M2) (Wang et al., 2024; Ding et al., 2025), exacerbates the inflammatory microenvironment and hampers effective tissue repair (Liao et al., 2023; Shuai et al., 2025). Despite significant advances in clinical treatments, including mechanical debridement and antibiotic therapy, the therapeutic outcomes of periodontitis remain limited. This is largely attributed to the persistent nature of the bacterial infection and the difficulty in effectively modulating the inflammatory microenvironment (Yu et al., 2026).

In recent years, bioactive materials have emerged as promising therapeutic strategies for periodontal regeneration (Yao et al., 2025). Among them, bioactive glass has garnered significant attention owing to its ability to stimulate bone formation and promote tissue repair (Zhou et al., 2024a; Li L. et al., 2025). However, conventional bioactive glass lacks intrinsic antibacterial activity and exhibit limited capability to modulate the immune response, which restricts its therapeutic efficacy (Hu W. et al., 2024; Ge et al., 2025). Copper, an essential trace element with well-known antimicrobial properties (Gao et al., 2023), has been shown to possess potent antibacterial activity against a broad spectrum of pathogens, including those associated with periodontitis (Wang Q. et al., 2025). Moreover, copper has been demonstrated to regulate immune responses by influencing macrophage polarization (Wen et al., 2025). Specifically, copper can promote the transition of macrophage from a pro-inflammatory M1 phenotype to an anti-inflammatory M2 phenotype (Pan S. et al., 2025), thereby creating a more favorable microenvironment for tissue repair (Zhang et al., 2025; Li et al., 2026). Therefore, copper-based biomaterials have a wide range of applications in antimicrobial protection and tissue repair. These materials include copper nanoparticles (Crisan et al., 2021), copper-based bio-coatings (Cieślik et al., 2025), and copper-doped composites (Munir et al., 2022; Li Y. et al., 2024), etc. Among them, incorporating copper into bioactive glass represents a promising strategy to simultaneously address two critical challenges in the periodontitis therapy, infection control and immune modulation.

To further enhance the clinical applicability of this material, we developed a copper-doped bioactive glass (Cu-BG) that can be crosslinked with hyaluronic acid (HA) through borate ester bonds to form an injectable hydrogel (Scheme 1). This hydrogel is designed to provide sustained release of copper ions, thereby exerting potent antibacterial effects against *S*. *aureus* and *P. gingivalis* while simultaneously modulating the macrophage polarization towards an anti-inflammatory M2 phenotype. Additionally, the hydrogel is expected to promote the osteogenic differentiation of mouse embryonic osteoblast precursor cells (MC3T3-E1), thereby facilitating periodontal tissue regeneration. Given these multifunctional therapeutic properties of this injectable hydrogel, we hypothesize that it will serve as an effective strategy for the treatment of periodontitis, potentially offering a significant advantage over current approaches.

**SCHEME 1:**
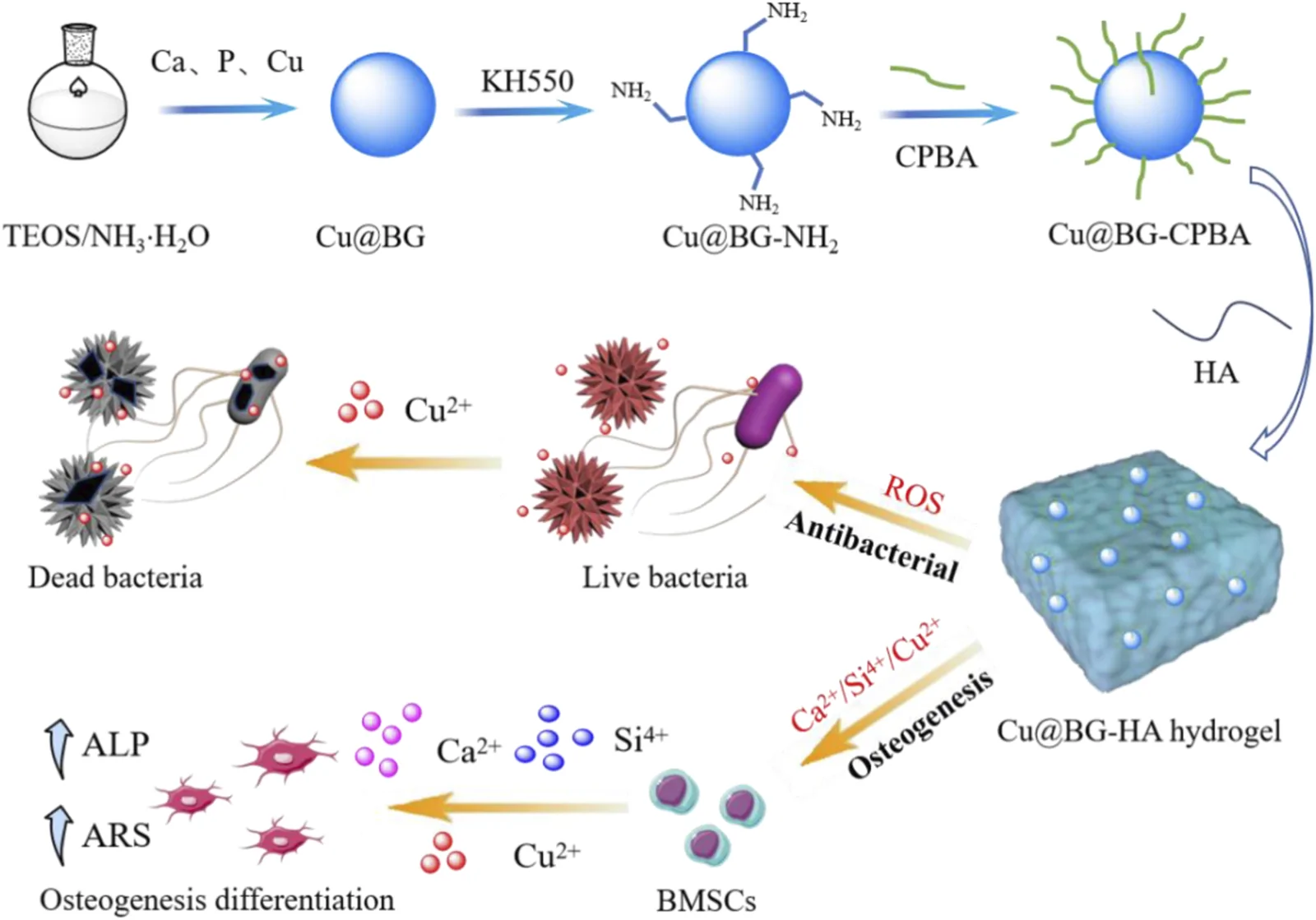
Schematic illustration of Cu@BG-HA hydrogel preparation and therapeutic mechanisms for periodontitis. Sol–gel synthesis of Cu-doped mesoporous bioactive glass nanoparticles with evaporation-induced self-assembly using CTAB as template. Surface functionalization: APTES-mediated amination followed by EDC/NHS-coupled CPBA grafting to yield phenylboronic-acid-modified Cu@BG. Dynamic borate ester crosslinking between Cu@BG and cis-diol groups on HA chains, forming an injectable hydrogel. Therapeutic mechanisms: sustained Cu^2+^ release exerts (i) bactericidal activity against periodontal pathogens; (ii) immunomodulation through LPS/IFN-γ-primed macrophage repolarization from pro-inflammatory M1 (iNOS) to anti-inflammatory M2 (CD206) phenotype; and (iii) osteogenic potentiation of MC3T3-E1 pre-osteoblasts via Runx_2_/OPN/OCN axis activation, synergistically driven by co-released Si^4+^, Ca^2+^, and PO_4_^3-^.

## 2 Materials and methods

### 2.1 Materials

Cetyltrimethylammonium bromide (CTAB), tetraethyl orthosilicate (TEOS), triethyl phosphate, calcium nitrate tetrahydrate, and 4-carboxyphenylboronic acid (CPBA) were purchased from Aladdin Reagent Co., Ltd. (Shanghai, China). Sodium hyaluronate (HA, 90 kDa) was supplied by Sinopharm Chemical Reagent Co., Ltd. (Shanghai, China). 3-Aminopropyltriethoxysilane (APTES), 1-ethyl-3-(3-dimethylaminopropyl)carbodiimide (EDC), and N-hydroxysuccinimide (NHS) were obtained from Sigma-Aldrich (MO, USA). Cell Counting Kit-8 (CCK-8), alkaline phosphatase (ALP) assay kit, BCIP/NBT staining kit, and alizarin red S (ARS) were provided by Beyotime Biotechnology (Shanghai, China). Fetal bovine serum (FBS) was purchased from Thermo Fisher Scientific (MA, USA). Anti-iNOS (rabbit) and anti-CD206 (mouse) primary antibodies were purchased from Beyotime Biotechnology (Shanghai, China). All reagents were used as received without further purification.

### 2.2 Synthesis of Cu-doped mesoporous bioactive glass nanoparticles (Cu-BG)

Cu-BG nanoparticles were prepared using an evaporation-induced sol–gel method adapted from previous reports (Dziadek et al., 2018). Briefly, CTAB (0.70 g) was dissolved in ultrapure water (100 mL) containing ethyl acetate (2 mL). The solution was adjusted to pH 10.5 using aqueous NH_3·_H_2_O (25%), after which TEOS (3.6 mL) was added under magnetic stirring (400 rpm, 30 °C). After 30 min, triethyl phosphate (0.36 mL), Ca(NO_3_)_2_·4H_2_O (2.277 g) and Cu(NO_3_)_2_·3H_2_O (0.145 g, 2 mol% Cu relative to Ca) were sequentially introduced at 30 min intervals. The mixture was stirred for 4 h and then aged overnight. The resulting precipitate was collected by filtration, washed with ethanol/water (1:1 v/v) and freeze-dried. The obtained powder was calcined at 600 °C (heating rate 2 °C min^-1^) for 5 h to remove CTAB. The final composition of the nanoparticles were SiO_2_-CaO-P_2_O_5_-CuO. The nanoparticles without copper ions were named BG.

### 2.3 Fabrication of Cu@BG-HA injectable hydrogel

Cu@BG nanoparticles (0.70 g) were dispersed in ethanol (200 mL) containing APTES (0.4 mL) and refluxed at 80 °C for 12 h to yield amine-functionalized BG-NH_2_. After centrifugation (9,000 × *g*, 10 min) and washing, the particles were re-dispersed in MES buffer (10 mL, 0.05 M, pH 6.5). Subsequently, CPBA (0.10 g) pre-activated with EDC (0.12 g) and NHS (0.08 g), was added to the suspension and stirred at 25 °C for 12 h to obtain phenylboronic-acid-grafted Cu@BG (Cu@BG-PBA). Cu@BG-PBA was mixed with HA (1.0 wt% in PBS, pH 7.4) at a volume ratio of 1:9. Dynamic borate-ester crosslinking occurred within 30 s, resulting in the formation of an injectable Cu@BG-HA hydrogel. In addition, BG-HA hydrogels were prepared using BG nanoparticles.

### 2.4 Physicochemical characterization

The morphology of nanoparticles and lyophilized hydrogels was examined by field-emission SEM (SU-8020, Hitachi, Japan) after gold sputter-coating. Cu^2+^ release was quantified by inductively coupled plasma optical emission spectroscopy (ICP-OES, Agilent 5110, USA) in PBS (pH 7.4, 37 °C) over 24 days. Fourier-transform infrared spectra (FTIR, Nicolet iS50, Thermo Fisher) were collected (4,000–400 cm^-1^). Thermogravimetric analysis (TGA, Netzsch, Germany) was performed under nitrogen (N_2_) atmosphere (10 °C min^-1^, 30 °C–800 °C). Rheological properties were measured using a MARS 3 rheometer (Thermo Haake, Germany) with a 25 mm parallel-plate geometry (0.1%–1,000% strain, 10 Hz, 25 °C).

### 2.5 Antibacterial properties

A single colony of *P. gingivalis* bacterial were cultured on brain heart infusion (BHI) agar and subsequently transferred to 10 mL liquid medium, followed by incubation at 37 °C for 24 h. Hydrogel extracts (50–200 µg mL^-1^) were prepared by incubating Cu@BG-HA in phosphate-buffered saline (PBS) at 37 °C for 24 h. For colony counting, 100 µL bacterial suspension (10^6^ CFU mL^-1^) was added to 900 µL extract and incubated at 37 °C for 6 h. Follwing 10^–5^ serial dilutions, aliquots were plated on BHI agar, and colonies were enumerated following 12 h incubation. Antibacterial efficacy (R) was calculated according to the following Equation 1:

$$R=\frac{A_{1}-A_{2}}{A_{1}}\times 100\%$$ (1)

where A_1_ and A_2_ are CFU of the control and treated groups, respectively.

*Staphylococcus aureus* were cultured in LB broth to mid-log phase (OD600 ≈ 0.6), the antibacterial efficiency was measured using the above method. In addition, kinetic growth curves were recorded in 96-well plates (90 µL extract + 10 µL inoculum), the optical density at 600 nm (OD_600_) was measured every 30 min for 12 h.

### 2.6 *In vitro* cytocompatibility

Mouse embryonic osteoblasts cells (MC3T3-E1) were purchased from Shanghai EK-Bioscience Biotechnology Co., Ltd. (Shanghai, China). MC3T3-E1 (1 × 10^4^ cells well^-1^) were cultured in α-MEM supplemented with 10% FBS and 1% penicillin–streptomycin for 24 h. Cells were then exposed to hydrogel extracts (25–150 µg mL^-1^) for an additional 24 h. Cell viability was assessed using CCK-8 (450 nm, Infinite 200 PRO, Tecan) and calculated as Equation 2:

$$Cell\;Viability\;\left(\%\right)=\frac{{OD}_{1}-{OD}_{2}}{{OD}_{0}-{OD}_{2}}\times 100\%$$ (2)

where OD_1_, OD_0_ and OD_2_ correspond to the optical density values of experimental groups, control groups and blank groups, respectively.

Live/dead staining was performed using acridine orange/ethidium bromide (AO/EB), and imaged by fluorescence microscopy (IX73, Olympus, Japan).

### 2.7 Osteogenic differentiation

MC3T3-E1 cells (1 × 10^4^ cells well^-1^) were cultured in osteogenic medium (α-MEM + 10% FBS, 50 µg mL^-1^ ascorbic acid, 10 mM β-glycerophosphate and 100 nM dexamethasone) containing different hydrogels extract (200 µg mL^-1^). ALP activity was quantified after 7 days using an ALP assay kit and normalized to total protein (BCA method). Mineralization was evaluated on day 14 by alizarin red S staining. Stained nodules were destained with 10% cetylpyridinium chloride, and the absorbance of the extracted solution was measured at 562 nm.

The expression levels of osteogenic marker genes were quantified by quantitative real-time polymerase chain reaction (qRT-PCR). After co-culturing MC3T3-E1 cells with the hydrogel for 7 days, total RNA was collected from the cells and reverse transcribed using PrimeScript master mix to obtain cDNA. Then, osteogenic-specific primers (GAPDH, OCN, OPN, Runx2) were used for qRT-PCR (primer sequences provided in Table 1). The gene expression levels of each experimental group were calculated using the 2^−ΔΔCt^ method.

**TABLE 1:** The primer sequence for polymerase chain reaction.

Gene	Primer sequence (5′-3′)
OCN	Forward: ACA AGT CCC ACA CAG CAA C Reverse: CCA GGT CAG AGA GGC AGA
OPN	Forward: GCGCATTACAGCAAACAC Reverse: TGGTCTCCGTCGTCATC
Runx-2	Forward: CGA ACA GAG CAA CAT CTC C Reverse: GTC AGT GCC TTC CTT GG
GAPDH	Forward: CAA GAA GGT GGT GAA GCA G Reverse: CAA AGG TGG AAG AAT GGG

### 2.8 Macrophage polarization assay

RAW264.7 cells were purchased from Shanghai EK-Bioscience Biotechnology Co., Ltd. (Shanghai, China). RAW264.7 (1 × 10^4^ cells well^-1^) were primed with LPS (1 µg mL^-1^) plus IFN-γ (20 ng mL^-1^) for 6 h, followed by treatment with different hydrogels extract (200 µg mL^-1^) for 24 h. After treatment, cells were fixed with 4% paraformaldehyde (15 min), permeabilized (0.1% Triton X-100, 10 min) and blocked (5% BSA, 1 h). Primary antibodies against iNOS (rabbit, 1:200) and CD206 (mouse, 1:200) were applied overnight at 4 °C, followed by Alexa Fluor 488-conjugated goat anti-mouse (1:500) and TRITC-conjugated goat anti-rabbit (1:500) secondary antibodies (2 h, room temperature). Nuclei were counterstained with DAPI. Images were acquired using an inverted fluorescence microscope (IX83, Olympus) and fluorescence intensity was quantified using ImageJ (NIH, USA).

The expression profiles of inflammation-related genes, including cluster of differentiation 206 (CD206), inducible nitric oxide synthase (iNOS), and tumor necrosis factor-alpha (TNF-α), were assessed using an identical experimental workflow. Total RNA extraction, reverse transcription, and qRT-PCR procedures were performed as described above for osteogenic gene analysis, with gene-specific primers substituted accordingly (primer sequences provided in Table 2). Relative expression levels were quantified using the 2^−ΔΔCt^ method with GAPDH as the endogenous reference gene.

**TABLE 2:** The primer sequence for polymerase chain reaction.

Gene	Primer sequence (5′-3′)
TNF-α	Forward: CGCTGAGGTCAATCTGC Reverse: GGCTGGGTAGAGAATGGA
CD206	Forward: ATCTCGCAAGCCGATGTACAC Reverse: ATCTCGCAAGCCGATGTACAC
iNOS	Forward: ATCCCGAAACGCTACACTT Reverse: CGGCTGGACTTCTCACTC
GAPDH	Forward: TGTTTCCTCGTCCCGTAGA Reverse: ATCTCCACTTTGCCACTGC

### 2.9 Statistical analysis

GraphPad Prism version 8.0.2 software (GraphPad Software, San Diego, CA, USA) was used to analyze the data. All data were presented as mean ± SD (standard deviation) and analyzed by Analysis of Variance (ANOVA) using differences with P values of less than 0.05 (*) indicated significance.

## 3 Results

### 3.1 Synthesis and physicochemical characterization of Cu@BG-HA hydrogel

Bioactive inorganic nanoparticles serve as functional modulators of hydrogel physicochemical properties (Wang et al., 2022). Herein, copper-doped bioactive glass nanoparticles (Cu@BG) were incorporated into HA matrices via dynamic borate ester crosslinking to fabricate an injectable, bioactive hydrogel. Scanning electron microscope (SEM) revealed that Cu@BG nanoparticles exhibited monodisperse spherical morphologies with a mean diameter of 480 ± 31 nm (Figure 1A). Following incorporation into the HA network, Cu@BG nanoparticles remained homogeneously distributed without macroscopic aggregation (Figures 1B,C), indicating effective matrix compatibility. Thermogravimetric analysis revealed a inorganic content of 29 wt% in Cu@BG-HA, coinciding with a residual mass after polymer decomposition (Figure 1D). FTIR spectra retained the characteristic of HA absorption bands at 3,280 cm^-1^ ν(O–H) and 1,615 cm^-1^ ν(C=O)) and displayed a new band at 1,370 cm^-1^ assigned to B–O stretching, confirming borate-ester cross-linking (Figure 1E) (Han et al., 2024; Zhang et al., 2020). Furthermore, Cu@BG-HA was loaded into a 1-mL syringe with 26G needle and extruded at controlled flow rates. The hydrogel exhibited smooth, continuous extrusion without clogging or fracture, confirming clinical injectability through periodontal pocket cannulae (Figure 1F). Rheological measurements showed that the storage (G′) and loss (G″) moduli were independent of strain between 0.1–1,000%, with G′ ≈ 1.2 kPa at 10 Hz, indicating a stable viscoelastic network suitable for injection (Figures 1G–I).

**FIGURE 1:**
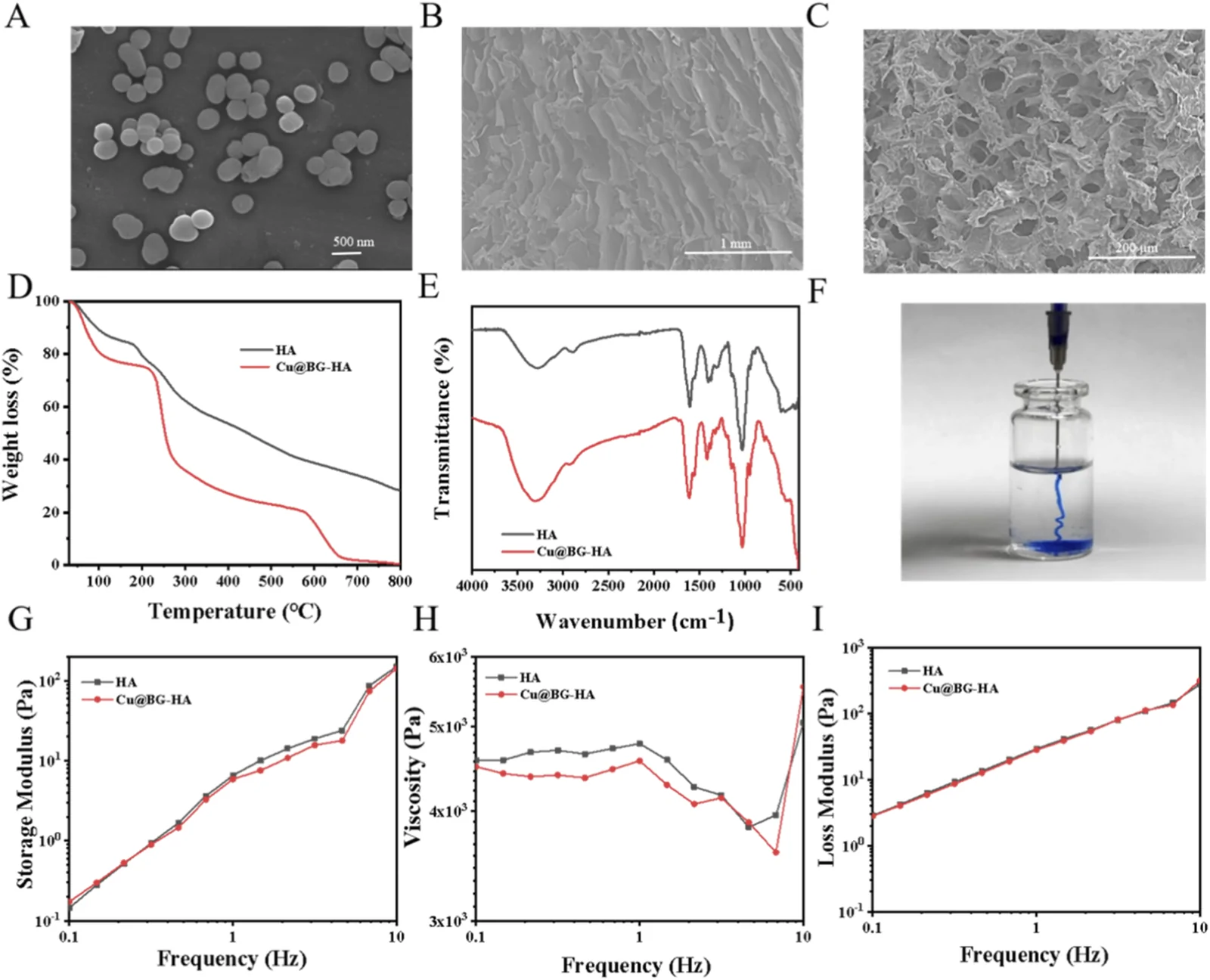
Characterization of hydrogel properties. SEM images of **(A)** Bioactive glass nanoparticles, **(B)** HA hydrogel and **(C)** Cu@BG-HA hydrogel. **(D)** Thermogravimetric analysis of HA hydrogel and Cu@BG-HA hydrogel. **(E)** FTIR spectra of HA hydrogel and Cu@BG-HA hydrogel. Rheological properties of hydrogels. **(F)** Image of hydrogel injectability in PBS. **(G)** Storage modulus **(H)** Viscosity and **(I)** loss modulus.

ICP-OES quantified a sustained Cu^2+^ release in a range of 10–100 µM over 24 days (Figure 2A), a range previously demonstrated to exert antibacterial activity without eliciting cytotoxicity towards periodontal cells. The hydrogel is capable of continuously releasing active ions such as silicon (Si), calcium (Ca), and phosphorus (P) in PBS solution, confirming that copper ions have been effectively incorporated into the amorphous covalent network structure of MBG (Figures 2B–D). Collectively, these results confirm the successful integration of Cu-BG into an injectable hydrogel while preserving its chemical integrity.

**FIGURE 2:**
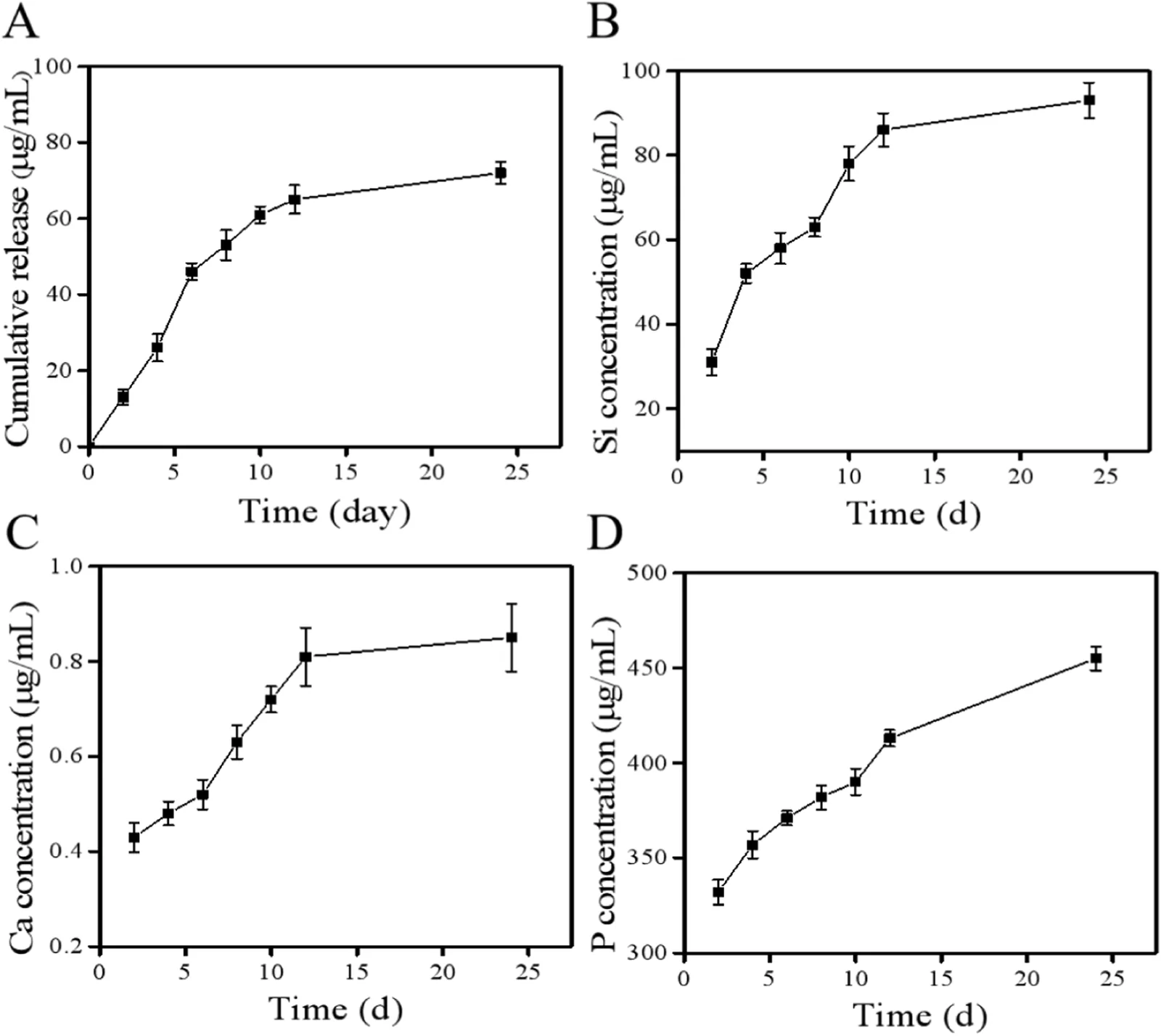
Ion release behavior of hydrogels. The concentration of **(A)** copper, **(B)** silicon, **(C)** calcium, and **(D)** phosphorus released form the hydrogels over time in PBS solution. Data represent mean ± SD; n = 3.

### 3.2 Cytocompatibility of Cu@BG-HA

HA acid and bioactive glass both exhibit promising potential for tissue engineering applications (Rethi et al., 2024). Cell viability assays following co-culture of MC3T3-E1 cells with hydrogel extract indicated that the HA hydrogel extract exerted no adverse effects on MC3T3-E1. Conversely, a modest, dose-independent proliferative trend was observed (Figure 3A). Cu@BG-HA extracts at concentrations ≤ 150 µg mL^-1^ preserved > 85% cell viability over 24 h, whereas the 200 µg mL^-1^ extract reduced metabolic activity to ∼78%, attributable to transient elevation in free Cu^2+^ (≈100 µM) at this concentration. Live/dead fluorescence staining corroborated these data: green AO fluorescence dominated across all Cu@BG-HA concentrations, with only sporadic red EB signals detect at the highest dose (Figure 3B). These findings are consistent with reported thresholds for Cu^2+^ in osteoblastic lineages, and confirm an acceptable therapeutic window for periodontal applications.

**FIGURE 3:**
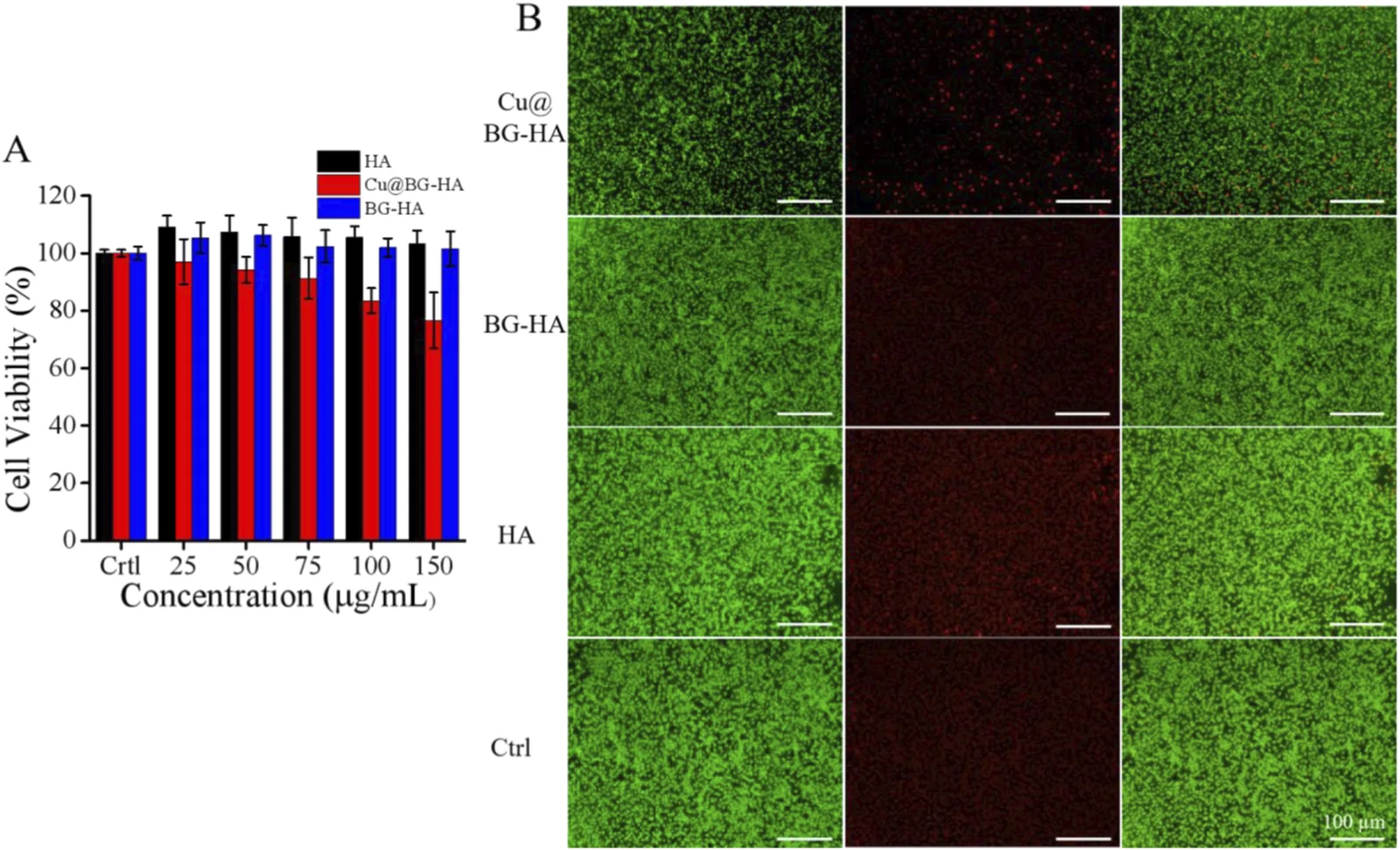
Cytocompatibility of hydrogels. **(A)** The cell viability and **(B)** fluorescence images of MC3T3-E1 cells after 24 h co-culture with different hydrogels. Data are presented as the mean ± SD; n = 3.

### 3.3 Antibacterial performance of Cu@BG-HA

Bioactive glass scaffolds support osteogenesis but lack intrinsic antibacterial activity, which can predispose to infection relapse (Wu et al., 2016). Copper ions exert bacterial effect by disrupting membrane integrity through electrostatic interaction with phospholipids and by oxidizing thiol-group, leading to metabolic arrest (Sobierajska et al., 2022). We therefore evaluated the dose-dependent bactericidal activity of Cu@BG-HA against periodontal pathogens. Against *Staphylococcus aureus*, the kill rate increase from 75.5% ± 6.6% at 50 µg mL^-1^ to 99.8% ± 3.2% at 100 µg mL^-1^ (Figure 4A), and no colonies were recoverable at 200 µg mL^-1^ (Figure 4C). Growth-kinetics measurements corroborated these data, with 100 µg mL^-1^ completely suppressing the exponential growth phase (Figure 4B). The steep concentration–response curve accords with a Cu^2+^-mediated mechanism and aligns with previously reported thresholds for mesoporous copper-doped bioactive glass (Gao et al., 2023). Notably, the bactericidal plateau (≥99%) is achieved at a Cu^2+^ release of ∼50 µM—well below the cytotoxic threshold for mammalian cells.

**FIGURE 4:**
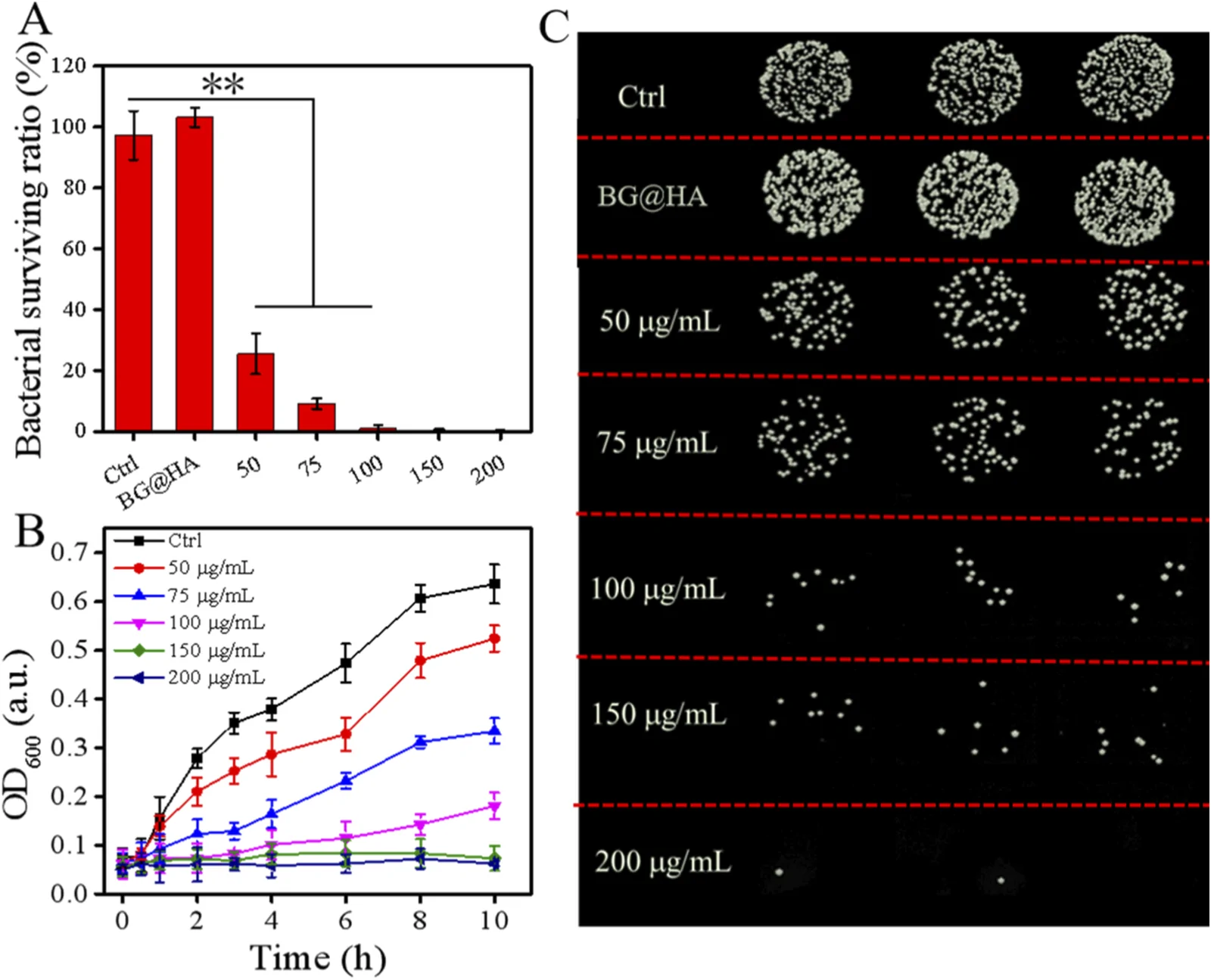
Antibacterial efficiency of **(A)** *Staphylococcus aureus* treated with various samples for 6 h. **(B)** Growth kinetic curves of *Staphylococcus aureus* treated with different concentration of Cu@BG-HA. **(C)** Representative images of *Staphylococcus aureus* colonies after 6 h treatment with Cu@BG-HA. Data are presented as the mean ± SD; n = 3; **p < 0.01.

Cu@BG-HA exhibited a right-shifted dose–response curve for *P. gingivalis*. At 50 and 75 µg mL^-1^, antibacterial efficiency remained below 50%, but increased sharply to 98.6% ± 1.7% at 150 µg mL^-1^ (Figure 5A). Growth-kinetic profiles mirrored this trend: with only the 150 µg mL^-1^ cohort entered stationary phase below the detection limit (Figure 5B). Compared with the BG-HA group, the introduction of copper ions effectively enhanced the hydrogel’s antimicrobial activity. Plate counts corroborated these findings, showing a 4.2-log_10_ reduction exclusively at the highest dose (Figure 5C). The EC_50_ against *P. gingivalis* (121 ± 6 µg mL^-1^) was 1.9-fold higher than that against *S. aureus*. The differential susceptibility is consistent with the structural disparity between Gram-positive and Gram-negative envelopes.

**FIGURE 5:**
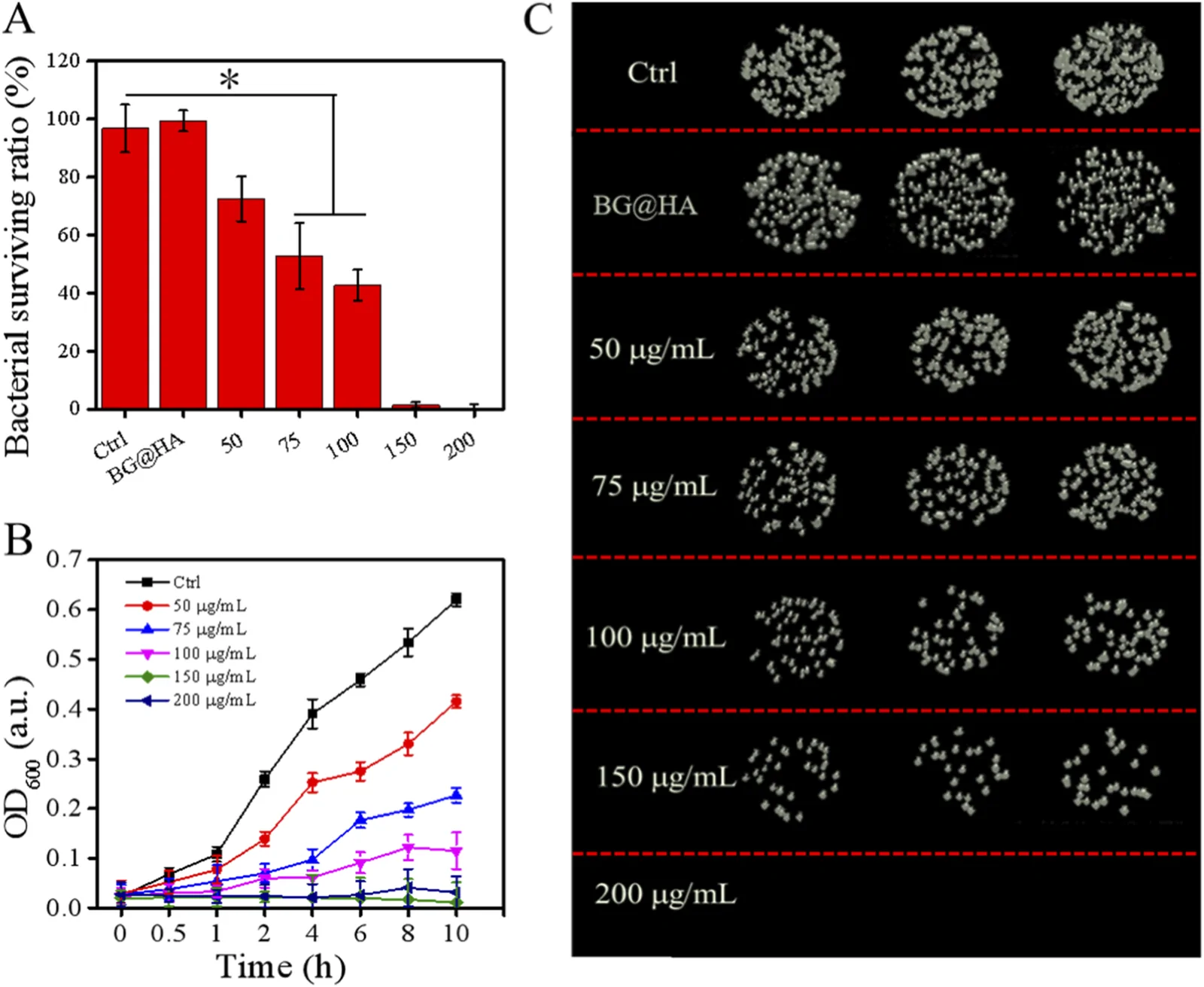
Antibacterial efficiency of **(A)** *Porphyromonas gingivalis* treated with various samples for 6 h. **(B)** Growth kinetic curves of *Porphyromonas gingivalis* treated with different concentration of Cu@BG-HA. **(C)** Representative colonies images of *Porphyromonas gingivalis* after 6 h treatment with Cu@BG-HA. Data are presented as the mean ± SD; n = 3; *p < 0.05.

### 3.4 *In vitro* osteogenic properties

Bioactive glasses degrade to release Ca^2+^, PO_4_^3-^, Si(OH)_4_, and trace Cu^2+^ ions that synergistically activate osteogenic signaling pathways (Zhou et al., 2024b). After 7 days of culture, MC3T3-E1 cells exposed to Cu@BG-HA (200 µg mL^-1^) displayed an intense ALP chromogenic reaction (Figure 6A). Quantitative assay revealed a 1.8 fold increase in normalized ALP activity compared to the vehicle control, whereas the HA scaffold induced only a marginal 1.1 fold increase, the biological active glass hydrogel without copper ions (BG-HA) also showed a 1.5 times increase (Figure 6C), demonstrating that Cu2+ provides an additive osteogenic stimulus beyond BG-derived ions. By day 21, extracellular matrix mineralization, an end-point marker of osteogenesis, was assessed by Alizarin Red S staining. Cu@BG-HA yielded densely packed, 1.7 fold higher than the control (Figures 6B,D), whereas HA again produced a modest 1.2-fold increase. Moreover, the expression of the osteogenic transcription factors Runx2, the osteogenic proteins OPN and OCN also indicated that the Cu@BG-HA hydrogel group exhibited the highest activity in promoting osteogenic differentiation (Figures 6E–G). Furthermore, compared with the BG-HA group, the addition of copper ions effectively promotes osteogenic differentiation in osteoblasts.

**FIGURE 6:**
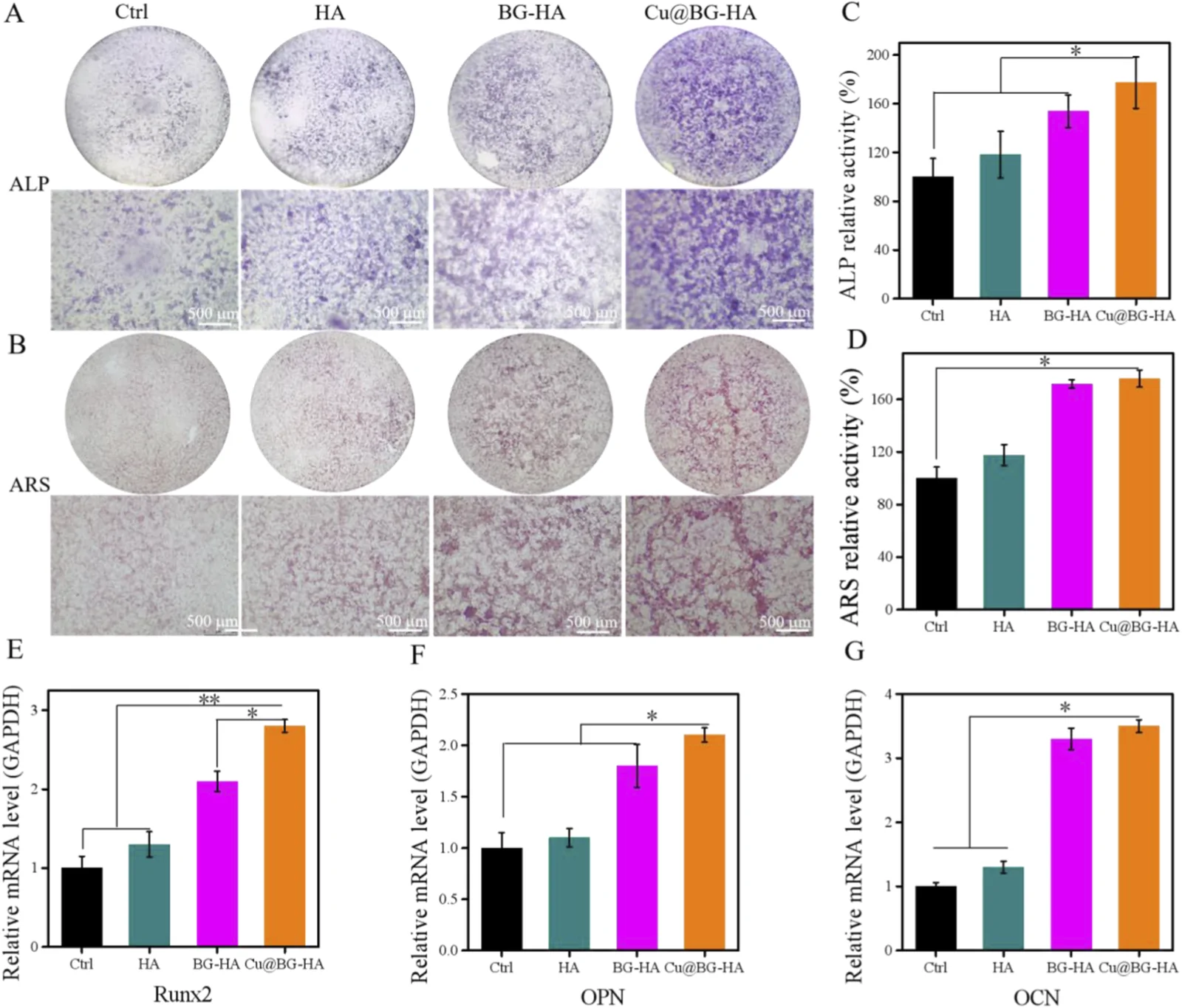
Osteogenic properties of hydrogels. **(A)** ALP and **(B)** ARS staining images of MC3T3 cells treated with HA, BG-HA and Cu@BG-HA. Quantitative analysis of **(C)** ALP activity and **(D)** ARS-stained mineralization. Expressions of osteogenic factors **(E)** Runx2, **(F)** OPN and **(G)** OCN. Data are presented as the mean ± SD; n = 3; *p < 0.05, **p < 0.01.

### 3.5 Immunomodulatory capacity of Cu@BG-HA hydrogel

Macrophage phenotype switching governs the progression of peri-implant inflammation and subsequent tissue regeneration (Huangfu et al., 2023). M1 macrophages (iNOS) amplify pro-inflammatory cascades, whereas M2 macrophages (CD206) secrete anti-inflammatory and pro-remodeling mediators (Ni et al., 2024). Copper ions have been reported to exert complex regulatory effects on the immune microenvironment of periodontitis through multiple mechanisms, including modulating immune cell activity, inducing copper-dependent cell death, intervening in signaling pathways, and regulating oxidative stress (Zhou et al., 2026). In this study, we utilized lipopolysaccharide (LPS) to stimulate macrophages and investigated the ability of Cu@BG-HA to regulate the transformation of M1 to M2 type macrophages through immunofluorescence. As shown in Figure 7A, under LPS-induced inflammatory conditions, the fluorescence intensity of CD206 in RAW264.7 cells was significantly higher in the Cu@BG-HA group than in the BG-HA and HA group, while iNOS fluorescence intensity was notably reduced. This indicates that the Cu@BG-HA hydrogel effectively promotes the M2 polarization and suppresses M1 macrophages. Quantitative analysis revealed a 1.27-fold increase in CD206 expression and 0.7-fold decrease in iNOS expression compared to the control (Figures 7B,C). These findings suggest that Cu@BG-HA hydrogel can induce macrophages polarization towards the M2 phenotype in an inflammatory environment, highlighting its significant anti-inflammatory potential. As shown in Figures 7D–F, the expression of anti-inflammatory factors CD206 was highest in the Cu@BG-HA group, whereas the expression of inflammatory factors INOS and TNF-α was lowest. Notably, BG-HA alone showed marginal or non-significant effects compared to pure HA controls in macrophage polarization assays (CD206: 1.27-fold vs. control, iNOS: 0.7-fold vs. control), confirming that the bioactive glass-derived ions do not substantially modulate immune phenotype in the LPS/IFN-γ priming model. In contrast, Cu@BG-HA elicited robust, statistically significant shifts in all measured parameters. These findings suggest that Cu^2+^ exerts a positive regulatory effect on anti-inflammatory factors. Therefore, copper-doped bioactive glass hydrogels exhibit significant anti-inflammatory effects.

**FIGURE 7:**
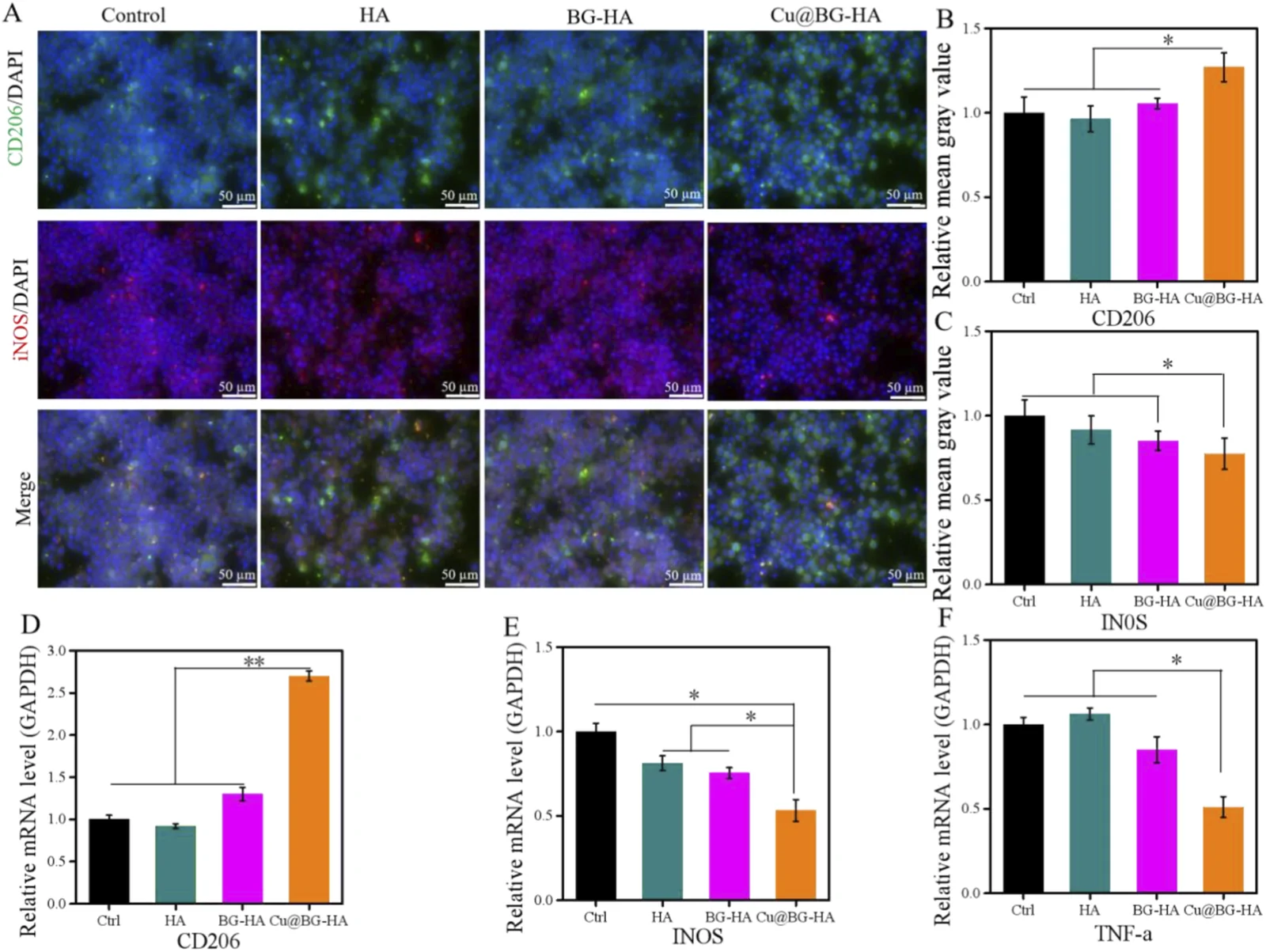
Hydrogel immunomodulatory properties. **(A)** Immunofluorescence staining images of RAW264.7 cells co-cultured with hydrogel. CD206: green, iNOS: red, nuclei: blue. Relative fluorescence expression level of **(B)** CD206 and **(C)** INOS. Expression of anti-inflammatory factors **(D)** CD206 and inflammatory factors **(E)** INOS, **(F)** TNF-α. Data are presented as the mean ± SD; n = 3; *p < 0.05, **p < 0.01.

## 4 Discussion

While Cu-BGs have been reported for antibacterial and osteogenic applications, existing studies predominantly rely on bulk-doping strategies that yield uncontrolled, burst-release kinetics incompatible with the chronic, recurrent nature of periodontitis (Lallukka et al., 2024). In our design, Cu^2+^ is precisely incorporated into mesoporous bioactive glass nanoparticles (480 nm) with tunable pore architecture (Figure 1A), enabling a sustained, zero-order-like release profile spanning 24 days within the narrowly defined therapeutic window of 10–100 µM (Figure 1I). This represents a critical advance over conventional Cu-BGs: The mesoporous architecture (synthesized via evaporation-induced sol–gel with CTAB template, calcined at 600 °C) ensures that Cu^2+^ release is governed by diffusion-limited transport from ordered nanopores rather than matrix degradation, decoupling ion availability from hydrogel dissolution kinetics. Furthermore, the boronic acid moiety serves a singular function as a reversible binding site. Our Cu@BG-HA system repurposes the borate ester bond as a multifunctional interface: (i) CPBA grafting onto Cu@BG-NH_2_: introduces surface-localized boronic acid density that crosslinks with HA diols to form a percolated nanoparticle–polymer network (Figure 1E, FTIR: 1,370 cm^-1^ B–O stretch); (ii) the dynamic nature of borate ester bonds (stable at physiological pH yet shear-thinning under injection) confers injectability without compromising network integrity (Figures 1F–H); and (iii) the nanoparticle-centric crosslinking topology positions each Cu@BG as a local ion-release node within the HA matrix, creating a spatially heterogeneous yet macroscopically uniform bioactivity distribution. This design achieves spatiotemporal matching between material properties and periodontal tissue dynamics: injectability for minimally invasive placement; sustained Cu^2+^ release for chronic infection management; and mechanical compliance matching native periodontal soft tissues.

The concentration-dependent antibacterial efficacy of Cu@BG-HA demonstrates favorable selectivity indices against periodontal pathogens. The differential susceptibility between *S. aureus* and *P. gingivalis* reflects fundamental structural disparities in bacterial envelope architecture. The thick peptidoglycan layer of *S. aureus* facilitates initial Cu^2+^ adsorption and penetration, whereas the lipopolysaccharide-rich outer membrane of P. gingivalis functions as a selective permeability barrier necessitating higher copper flux for effective disruption (Huang et al., 2025). This mechanistic insight suggests that optimal dosing regimens may require pathogen-specific calibration in polymicrobial periodontal infections. In this study, Cu@BG-HA platform targets the inflammatory-osteolytic triad (infection, immune dysregulation, bone loss) common to both periodontal disease and peri-implantitis. *Staphylococcus aureus* represents a critical nosocomial pathogen in implant-related infections (Kurz et al., 2024), and its robust peptidoglycan layer serves as a stringent test for Cu^2+^-mediated membrane disruption-thereby validating the broad-spectrum potential of our therapeutic approach. Subsequent studies will establish P. gingivalis–dominant biofilms, F. nucleatum-P. gingivalis dual-species biofilms, and *ex vivo* saliva-derived polymicrobial biofilms to validate Cu@BG-HA efficacy under anaerobic, matrix-enclosed conditions that more faithfully replicate the subgingival niche.

The repolarization of LPS/IFN-γ-primed macrophages from pro-inflammatory M1 toward anti-inflammatory M2 phenotypes represents a critical therapeutic mechanism for resolving periodontitis-associated inflammation. The observed 1.27-fold upregulation of CD206 and concomitant 0.70-fold downregulation of iNOS suggest that Cu^2+^ modulates the transcriptional programs governing macrophage polarization. The 1.8-fold enhancement in alkaline phosphatase activity and 1.7-fold increase in mineralized nodule formation demonstrate that Cu@BG-HA potentiates osteogenic differentiation through enhanced activation of canonical osteoblast differentiation programs (Li J. et al., 2025). BG-HA alone showed no significant effect on macrophage polarization (CD206: 1.05-fold; iNOS: 0.85-fold versus control), confirming that Cu^2+^ is the principal immunomodulatory effector.

Several limitations of this study should be acknowledged. First, all functional assessments were conducted *in vitro*; the pharmacokinetics of Cu^2+^ release, tissue integration, and therapeutic efficacy *in vivo* remain to be established. Second, the long-term biocompatibility and potential systemic copper accumulation following periodontal injection require evaluation in large-animal models. Third, the relative contributions of released Cu^2+^ versus intact nanoparticles to the observed biological effects warrant further mechanistic dissection through pathway-specific inhibition studies. While the present study establishes proof-of-concept for synchronized antibacterial-immunomodulatory-osteogenic activity *in vitro*, we acknowledge that pharmacokinetic validation, tissue integration, and therapeutic efficacy in large-animal periodontitis models are prerequisite steps toward clinical translation.

## 5 Conclusion

In this study, we developed an injectable Cu@BG–HA hydrogel capable of sustainably releasing 10–100 µM Cu^2+^ over 24 days. This hydrogel eradicated over 99% of *S. aureus* and *P. gingivalis* at concentrations of 150–200 µg mL^-1^, while concurrently repolarizing LPS/IFN-γ-primed macrophages from the pro-inflammatory M1 phenotype to the anti-inflammatory M2 phenotype. Additionally, it enhanced the ALP activity and mineralization of MC3T3-E1 cells by 1.8- and 1.7-fold, respectively. Collectively, these findings demonstrate that a single bioactive ion-delivery platform can synchronize antibacterial defense, immune resolution and osteogenesis a challenge that remains unmet in current periodontitis therapies. This study establishes Cu-doped mesoporous bioglass as a multi-functional, immunomodulatory, and pro-regenerative tool, offering a minimally invasive alternative to conventional mechanical-plus-antibiotic regimens. Future work will focus on developing a validated large-animal periodontitis model to correlate local Cu^2+^ pharmacokinetics with alveolar-bone regeneration.

## Data Availability

The original contributions presented in the study are included in the article/supplementary material, further inquiries can be directed to the corresponding author.
